# Multimodal Handheld Probe for Characterizing Otitis Media — Integrating Raman Spectroscopy and Optical Coherence Tomography

**DOI:** 10.3389/fphot.2022.929574

**Published:** 2022-06-17

**Authors:** Guillermo L. Monroy, Sean T. Fitzgerald, Andrea Locke, Jungeun Won, Darold R. Spillman, Alexander Ho, Farzana R. Zaki, Honggu Choi, Eric J. Chaney, Jay A. Werkhaven, Kevin M. Mason, Anita Mahadevan-Jansen, Stephen A. Boppart

**Affiliations:** 1 Beckman Institute for Advanced Science and Technology, University of Illinois Urbana-Champaign, Urbana, IL, United States; 2 Vanderbilt Biophotonics Center, Nashville, TN, United States; 3 Dept. Biomedical Engineering, Vanderbilt University, Nashville, TN, United States; 4 Dept. Bioengineering, University of Illinois Urbana-Champaign, Urbana, IL, United States; 5 Dept. Otolaryngology, Vanderbilt University Medical Center, Nashville, TN, United States; 6 Center for Microbial Pathogenesis, The Abigail Wexner Research Institute Nationwide Children’s Hospital, Columbus, OH, United States; 7 Dept. Electrical and Computer Engineering, University of Illinois Urbana-Champaign, Urbana, IL, United States; 8 Carle Illinois College of Medicine, University of Illinois Urbana-Champaign, Urbana, IL, United States; 9 Cancer Center at Illinois, University of Illinois Urbana-Champaign, Urbana, IL, United States

**Keywords:** otitis media, optical coherence tomography, Raman spectroscopy, handheld probe, middle ear fluid

## Abstract

Otitis media (OM) is a common disease of the middle ear, affecting 80% of children before the age of three. The otoscope, a simple illuminated magnifier, is the standard clinical diagnostic tool to observe the middle ear. However, it has limited contrast to detect signs of infection, such as clearly identifying and characterizing middle ear fluid or biofilms that accumulate within the middle ear. Likewise, invasive sampling of every subject is not clinically indicated nor practical. Thus, collecting accurate noninvasive diagnostic factors is vital for clinicians to deliver a precise diagnosis and effective treatment regimen. To address this need, a combined benchtop Raman spectroscopy (RS) and optical coherence tomography (OCT) system was developed. Together, RS-OCT can non-invasively interrogate the structural and biochemical signatures of the middle ear under normal and infected conditions.In this paper, *in vivo* RS scans from pediatric clinical human subjects presenting with OM were evaluated in parallel with RS-OCT data of physiologically relevant *in vitro* ear models. Component-level characterization of a healthy tympanic membrane and malleus bone, as well as OM-related middle ear fluid, identified the optimal position within the ear for RS-OCT data collection. To address the design challenges in developing a system specific to clinical use, a prototype non-contact multimodal handheld probe was built and successfully tested *in vitro*. Design criteria have been developed to successfully address imaging constraints imposed by physiological characteristics of the ear and optical safety limits. Here, we present the pathway for translation of RS-OCT for non-invasive detection of OM.

## 1 INTRODUCTION

Otitis media (OM) is one of the most common diagnoses for children under the age of three (Granath, 2017), and is the leading cause of outpatient antibiotic use for children (Klein, 2000). Side effects related to OM infections often result in bouts of sleepless nights, discomfort, lost time at school for children, and subsequently lost work hours for parents. This loss of time and resources results in approximately twenty-five million physician visits and $4.3 billion USD annually (Elden and Coyte, 1998; Tong et al., 2018) of socioeconomic financial impact in the United States alone. Appropriate clinical management, including early diagnosis, timely vaccination, and administration of antibiotics, prevents more severe complications for OM (e.g., abscess, death), which are typically rare in developed nations (Kacmarynski et al., 2004; Penido Nde et al., 2016). Therefore, a primary focus in the management of OM in children is to maintain hearing acuity for unimpeded speech and language development (Spaw and Camacho, 2021).

This disease is often caused by an upper respiratory-tract infection (URI) where inflammation in the nasopharynx can lead to dysfunction and blockage of the Eustachian tube, a normally aerated drainage route for the middle ear. This blockage leads to fluid accumulation in the ear (i.e., effusion), and often results in bacterial colonization of the middle ear cavity; most commonly *S. pneumoniae*, (non-typeable) *H. influenzae*, and *M. catarrhalis* strains (Gaddey et al., 2019). OM can be thought of more as an umbrella term for a distribution of related symptoms, which progress through distinct phases of severity. Acute OM (AOM) follows the body’s immediate response to bacterial infection, often identified by erythema (i.e., redness) and bulging of the tympanic membrane (TM). Fluid buildup in the middle ear cavity with no apparent acute symptoms is referred to as OM with effusion (OME). Apart from watchful waiting, broad-spectrum antibiotics are the standard treatment for AOM, and are reasonably effective (Gaddey et al., 2019). Recurrent AOM is classified as multiple bouts of infection in a short period (i.e., three infections in 6 months) (Wald, 2021), and can be treated with a stronger broad-spectrum antibiotic (Granath, 2017). If problems persist, surgical placement of tympanostomy tubes (TT, drainage grommets) under anesthesia is typically effective in clearing infection (Steele et al., 2017).

Biofilms have been theorized to be the cause of recurrent ear infections (Bakaletz, 2007; Silva and Sillankorva, 2019). Biofilms are collections of bacteria that have changed their genetic conformation to live in a community within harsh environments like the human body (Donlan and Costerton, 2002) and excrete a protective extracellular polymeric matrix (EPS) to survive. The EPS protects the encased bacteria from the host immune system (Hall-Stoodley et al., 2004), and likewise from being efficiently cleared by antibiotics. Many diseases are now thought to be caused by biofilms (Lebeaux et al., 2014)—including cystic fibrosis, dental plaques, urinary tract infections, and, more recently recurrent AOM. Our group has confirmed that the presence of additional layers adhered to the TM and middle ear mucosa are indeed active biofilms (Monroy et al., 2018), which presumably exist throughout the middle ear cavity. Biofilm presence in recurrent AOM may explain the repeated episodes and virulence of infection, and why placement of tubes assists in clearing this biofilm by draining any fluid and restoring aeration to the middle ear cavity (Spaw and Camacho, 2021). Identifying the presence or absence of biofilms during the diagnostic process could ensure antibiotics are more appropriately prescribed and reduce the related risk of increased antibiotic resistance (Novotny et al., 2019).

Diagnosing OM is performed most commonly using an otoscope (Niermeyer et al., 2019) to observe the TM for signs of OM infection (Sundvall et al., 2019), and to track the duration and severity of infection. The otoscope and speculum are used to help navigate through the ear canal and provide a view of the TM surface. The physician must interpret these visual cues of infection and determine a best course of action in accordance with recommended clinical guidelines for OM (Lieberthal et al., 2013). As a result, pediatrician accuracy to distinguish different infection states with otoscopy can widely vary from 47–93% (Pichichero and Poole, 2001), as the measures are interpreted subjectively based on individual experience. Other diagnostic tools like pneumatic otoscopy and tympanometry provide more precise and quantitative information about the mobility of the eardrum or altered middle ear pressure due to the presence of effusion. However, in practice, these tools are used much less frequently in clinical exams (Sundvall et al., 2019) due to the difficulty in achieving an air-tight seal in the ear canal. Recent surveys show that the vast majority of general practitioners and specialist otolaryngology trainees still primarily rely on otoscopy for evaluation of OM, while the use of other techniques varies significantly (Sundvall et al., 2019).

Optical imaging and sensing techniques are well suited to diagnose ear infections. The eardrum is a thin membrane, at ~80–100 μm thick in human adults (Van der Jeught et al., 2013), separating the ear canal and the middle ear space. AOM presents with inflammation and sometimes sterile fluid, OME with purulent fluid, and recurrent AOM with biofilm and fluid. Several advanced techniques and devices have been developed that probe these aspects of OM–such as better effusion detection through acoustic reflectometry (Muderris et al., 2013), “shortwave” light (Carr et al., 2016) or terahertz light (Ji et al., 2016), or by tracking TM mobility precisely using vibrometry (Kim et al., 2019). A comprehensive review of devices that can assess the ear for OM can be found in literature (Locke et al., 2020; Prasad et al., 2020). While many of these non-invasive optical characterization methods exist, no single technique provides a comprehensive picture of the infection state, which can capture both the structural and biochemical changes of the tissue (e.g., inflammation of TM, fluid presence and composition, presence of a biofilm, etc.).

To address this challenge, our team developed combined Raman spectroscopy (RS) and optical coherence tomography (OCT) systems for various diseases (Patil et al., 2008), and recently for OM (Zhao et al., 2016). As the optical analogue to ultrasound imaging, OCT collects cross-sectional depth scans of the TM and adjacent middle ear space by interferometric detection of back-scattered light (Huang et al., 1991) in place of reflected sound waves. Analysis of OCT images can quantify the structural changes present in middle ear tissues, such as the presence of any middle ear fluid, its texture or scattering properties, any thickening of TM layers due to inflammation, or added layers when compared to normal (e.g., presence of a TM-adherent biofilm). To validate the utility of OCT in otology, multiple studies have been performed to connect image features (Monroy et al., 2019) to specific infection states (Monroy et al., 2017), validate the presence of biofilms in the middle ear (Monroy et al., 2018), and track the effects of antibiotic therapy (Won et al., 2021a). While these metrics are of clinical significance to OM diagnostics, OCT lacks specificity in assessing biochemical changes. Examples include effusion composition of serous or mucoid fluid (i.e., glycoproteins, bacteria, neutrophils, leukocytes) (Schachern et al., 2017), or identification of the causative bacterial strains (Shen et al., 2022). One of the most commonly used optical chemical analysis methods to assess and quantify biological changes in tissues non-invasively is RS, a vibrational spectroscopy technique (American National Standard Inistitute, 2014; Szymanski et al., 2022). By analyzing the Raman spectrum of inelastically scattered light, RS allows for the characterization of the unique spectral fingerprint of tissues or biochemical changes within tissues associated with infection or disease. Our team has recently shown that the most common bacterial species responsible for OM can be distinguished using Raman microspectroscopy *in vitro* (Ayala et al., 2017). Subtle differences between mucoid and serous effusion types have been identified using RS (Pandey et al., 2018) when aspirated from the middle ear space.

Given the potential diagnostic improvement by using a combination of OCT imaging and RS, there is motivation to develop an appropriate and sensitive device for *in vivo* characterization of OM. Integration of a multimodal system for *in vivo* tissue characterization has previously been tested for applications in cancer diagnostics (Patil et al., 2011b; Mazurenka et al., 2017) and oral tissue identification (Wang et al., 2018). To this aim, we previously combined a custom-built RS fiber probe and 1D (non-scanning) OCT system to observe *ex vivo* tissue and concentrated bacterial broth (Zhao et al., 2016). Initial feasibility was demonstrated to detect bacterial signatures through a fresh *ex vivo* rat TM. More recently, the specific signatures of *in vitro* otopathogenic cultures and biofilms were characterized with RS and OCT (Locke et al., 2021).

However, translating such technologies for clinical use presents unique design challenges and physiological constraints, particularly for RS detection of middle ear component signals, which must be addressed before RS-OCT can successfully be applied for OM diagnosis *in vivo*. Acquiring Raman signals directly from the middle ear with a fiber optic RS probe is challenging because direct contact with the delicate TM is not possible. Compared to contact-based measurements, implementing RS in a lens-less stand-off configuration decreases collection efficiency and requires more precise axial positioning to maintain signal-count consistency between measurements. Otoscopy alone is insufficient to achieve a repeatable probe-tissue separation distance. Apart from the microstructural characterization of tissue, OCT can also provide this needed positioning feedback for RS measurements. Thus, applying multimodal RS-OCT in the middle ear provides both microstructural and biochemical characterization of observed tissues, as well as improved positioning and collection efficiency for RS.

This paper will address the specific challenges associated with implementing RS-OCT for *in vivo* observation and determine the physiological origins and technical constraints of multimodal signal collection within the middle ear. Specifically, the feasibility and challenges of acquiring Raman spectra from middle ear fluid was investigated in a clinical setting. In parallel, a custom-built RS and OCT platform was used to characterize OM-related factors in both *ex vivo* tissue and an *in vitro* infection model. A multimodal RS-OCT prototype was then fabricated to test the integrated hardware using realistic design parameters and constraints for the ear and OM. Lastly, maximum permissible exposure (MPE), optical safety limits, and their importance to clinically focused system designs are discussed.

## 2 METHODS

### 2.1 Imaging and Spectroscopy System

**FIGURE 1 |:**
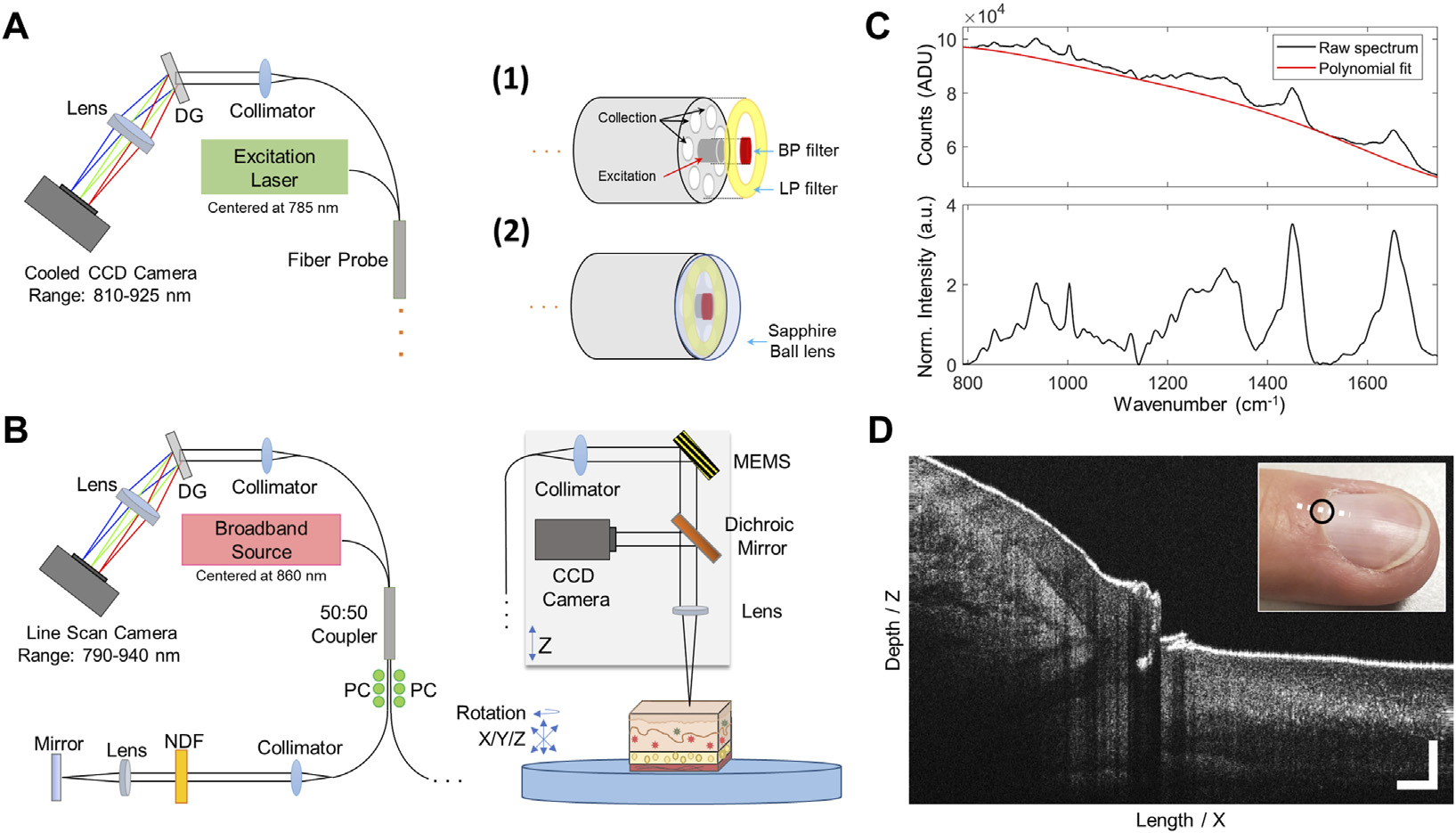
RS and OCT imaging systems with representative data. **(A)** RS benchtop system schematic and two custom-designed interchangeable RS probes. The probe used for clinical measurements (RS_1_) was lensless, while an equivalent probe with added sapphire ball lens (RS_2_) was used for *in vitro* measurements. **(B)** OCT benchtop system and sample stage for stable positioning of the sample. A color CCD camera collects surface images of the sample. **(C)** Representative sample data taken from the cuticle of a human fingernail. Raw (unprocessed) and final pre-processed Raman spectrum. **(D)** OCT data shows a cross-sectional image of the cuticle and nail bed. OCT scale bars represent 250 μm. DG, Dispersion grating; BP, bandpass; LP, longpass; NDF, Neutral density filter; PC, Polarization controller; CCD, charge-coupled device; MEMS, micro-electromechanical system 2-axis scan mirror

Representative diagrams of the systems used in this study are shown in Figure 1. A range of system configurations were considered using similar hardware currently employed for recent and ongoing RS (Unal et al., 2019; Rekha et al., 2020) and OCT (Won et al., 2021a; Won et al., 2021b) *in vivo* clinical studies. These systems were modified for use in this study to determine design parameters and test the theoretical maximum performance for RS-OCT in the middle ear.

#### 2.1.1 Raman Spectroscopy System and Calibration

A portable RS detection system with a 785 nm laser diode (II0785MU0350MS, Innovative Photonic Solutions, NJ), and two fiber-based optical probes were used to acquire Raman spectra (Figure 1A). The detection system includes an f/2.2 imaging spectrograph (HT Raman spectrometer, EmVision, LLC, FL) using a thermoelectrically cooled, deep depleted CCD camera (Blaze 400HR, Teledyne Princeton Instruments, CA) to acquire spectral data between 810–925 nm (i.e., 400–1900 cm^−1^) with a spectral resolution measured to be approximately 8 cm^−1^.

For *in vivo* RS measurements of the middle ear, a custom-designed lens-less fiber probe (Probe 1, EmVision, LLC., FL) was used. The probe design includes a single 300-micron core laser delivery fiber (0.22 numerical aperture, NA) surrounded by a ring of seven 300-micron core (0.22 NA) collection fibers to couple back-scattered emissions to the spectrometer. The excitation fiber is covered with a 785 nm laser line bandpass filter to block extraneous wavelengths from reaching the sample. An annular-shaped 800 nm longpass filter is set on top of the collection fiber ring to block the excitation laser line. For *in vitro* RS measurements of middle ear components, a ball-lens probe with similar construction to the clinical probe was used (Probe 2, EmVision LLC, FL). This RS probe consists of seven 300-micron core collection fibers, with a 3 mm diameter sapphire ball lens affixed to the front of the Raman fiber bundle (Figure 1A), providing a ~500 μm spot size at a 250 μm working distance. This probe was used to optimally characterize the RS signal from individual *ex vivo* middle ear components, As this probe is primarily used in near-contact with the tissue, it served as a performance benchmark in terms of maximized signal quality and spatial specificity for comparison to *in vivo* measurements taken later using the lens-less clinical probe.

Spectrometer calibration was performed prior to all RS measurements. Wavelength dispersion was calibrated using atomic emission lines from a neon-argon lamp. Relative Raman wavenumber shift was then calibrated with acetaminophen and naphthalene standard materials. System intensity response was calibrated using a NIST-traceable quartz-tungsten-halogen lamp (63976-45Q-OA, Newport, CA). The spectral pre-processing routine included binning of data to ½ spectral resolution (16 cm^−1^) and noise smoothing with a 2^nd^ order Savitzky-Golay filter. Following this, the background tissue autofluorescence was estimated with a modified polynomial fitting approach (Lieber and Mahadevan-Jansen, 2003) and subsequently subtracted. For measurements that were compared by normalized intensity, spectra were normalized to the mean intensity of the signal. An example from a human nail cuticle is shown in Figure 1C (scanned at the black circle), with raw RS signal (top) and final pre-processed spectrum (bottom panel) shown.

#### 2.1.2 Optical Coherence Tomography Imaging System

A custom-built benchtop OCT system (Figure 1B) employed in this study uses a broadband light source with a central wavelength of 860 nm and a bandwidth of ~145 nm, providing an axial resolution of 2.3 μm. The imaging beam had an optical power of ~2.35 mW at the sample. The objective lens produces a spot size of ~35 μm in diameter at the focus, in air. A spectrometer (Wasatch Photonics, NC, United States) and 4096-pixel line scan camera (Piranha 4, Teledyne DALSA, Ontario, CA) captures single depth profiles (A-lines) at a 30 μs integration time. A two-axis MEMS scan mirror (Mirrorcle, Inc., CA) was used to collect cross-sectional images and volumes at ~30 frames per second with 1000 A-lines per image. Color CCD images (XIMEA, CO, United States) of the sample surface were acquired during OCT imaging to monitor lateral scan position. The scan range on the benchtop configuration is adjustable and has a maximum range of ~8 mm × 4.5 mm × 8 mm, (Length/X × Depth/Z × Width/Y). Example OCT data from this system is shown in Figure 1D (white dotted line), centered at the same nail cuticle region measured from the RS system.

### 2.2 Clinical Raman Spectroscopy Acquisition–Recruitment and Protocol

An RS feasibility study was conducted at Monroe Carell Jr. Children’s Hospital under a protocol approved by the Vanderbilt University Medical Center Institutional Review Board (IRB no. 160263,161563). A total of 10 pediatric (6 m.o.—10 y.o.) human subjects that were scheduled for bilateral myringotomy and tympanostomy tube (TT) placement were enrolled in this study. Informed and written consent was obtained from the patient and legal guardian(s).

As part of the standard of care surgical procedure, each subject was brought into the operating room and placed under anesthesia. The surgeon cleared each ear canal of any obstructing earwax. Immediately prior to myringotomy, the lensless RS clinical probe was inserted through an ear speculum (Farrior, Integra LifeSciences, NJ, United States) and manually positioned at roughly 8 mm offset from the TM under visual guidance from the surgical microscope. The chosen probe-tissue distance offered a reasonably achievable compromise between signal collection efficiency and maintaining a safe probe-tissue offset to avoid damaging middle ear tissues.

Room lights were turned off temporarily and RS measurements were acquired. Four measurements were taken by manually aiming the probe at each major quadrant of the TM. Each acquisition took 6 s (2s exposure time, 3x accumulations) at 80 mW of power (measured at the probe tip). The diverging excitation source from the RS probe forms an illumination spot with approximately 3.6 mm diameter (1/e beam diameter width) on the tissue surface. After RS spectral acquisition, room lights were turned on, myringotomy was performed, and the presence (Effusion +) or absence (dry ears, Effusion −) of fluid was confirmed with the surgical microscope. If present, any effusion was aspirated from the middle ear, collected in a fluid trap, and stored at −80°C for later analysis. TT placement was subsequently performed as per standard of care. This procedure was then repeated in the contralateral ear. In total, the RS acquisition procedure added approximately 4 min (2 min/ear) to the surgical procedure and anesthesia time. The physician’s diagnosis was used as the gold standard for the determination of OM. No OCT scans were acquired for this portion of the study.

### 2.3 Development and Characterization of *in vitro* Otitis Media Models

#### 2.3.1 Animal Handling and Sample Collection

Intact TMs and ossicles were collected from two juvenile outbred chinchillas. The chinchilla is considered the goldstandard animal model for OM, as the anatomical dimensions and OM etiology are similar to humans (Bakaletz, 2009). Chinchillas were acquired from an approved commercial breeder (Rauscher Ranch, LaRue, OH) and animal experiments were completed in adherence with the *Guide for the Care and Use of Laboratory Animals* of the National Institutes of Health, and approved by the Institutional Animal Care and Use Committee (IACUC) at the Abigail Wexner Research Institute at Nationwide Children’s hospital in Ohio, United States (Welfare Assurance Number A35544-01). The chinchillas were socially housed in groups of three with corncob bedding in a biosafety level 2 facility. Experimental procedures were performed under anesthesia and all efforts were made to minimize any potential suffering. Animals were sacrificed and dissected to remove superior and inferior bullae. Two sets of TMs and ossicles were retrieved and placed in sterile saline, put onto dry ice, and shipped to Vanderbilt University where they were placed in −80°C freezer storage for later experimentation.

#### 2.3.2 Characterization of Chinchilla TM

To investigate the spatial distribution of RS and OCT signals from an isolated TM, a chinchilla TM was thawed to room temperature and suspended in a Petri dish filled with de-ionized water, then carefully transferred flat onto a Raman-grade Calcium Fluoride (CaF 2) slide (Crystran, Poole, United Kingdom) to minimize signal interference from the substrate. Once stable on the slide, RS scans were collected from the light reflex, umbo, and pars flaccida regions on the TM (Volandri et al., 2011). Measurements were obtained using the ball lens RS probe using 45 mW of power, measured at the probe tip. Spectra were acquired using 500 ms exposure time and averaged over five accumulations. The RS probe was mounted in a custom chuck and brought into near-contact (i.e., <1 mm) with the TM. Five spectra were acquired from each region by removing and replacing the probe to investigate signal reproducibility. For OCT scans, the focus of the system was placed just below the TM surface and scans were acquired from the same locations where RS scans were taken. Co-registration between measurements was ensured using structural landmarks, using the color CCD and translation stage in the OCT system for fine positioning. The tissue was kept hydrated during experiments using de-ionized water.

#### 2.3.3 Middle ear Model Construction and Signal Acquisition

An *in vitro* component model was created to closely simulate a possible clinical presentation of OME. First, a mucoid human effusion sample (~ 1 ml) was thawed to room temperature and an aliquot was deposited onto a CaF_2_ slide. A resected chinchilla TM with adhered malleus was then thawed to room temperature and gently placed over the effusion sample. Raman spectra from the resultant OME model were measured with the ball-lens RS probe at several locations to determine the maximum feasible signal that could be collected under controlled conditions. The RS probe was mounted for stability in a custom chuck and brought into near-contact (i.e., < 1 mm) over three regions: 1) umbo overlaid effusion, 2) light reflex of TM overlaid the effusion, and 3) effusion alone. Identical laser power and acquisition settings (45 mW, measured at the probe tip, 500 ms exposure time, five accumulations) to the TM measurements were used. After each RS measurement, the sample was transferred to the OCT system and imaged over the same region where RS scans were acquired. The focus of the OCT system was placed just below the surface of the model to center the tissue layers within the imaging range. As before, measurements were co-registered using structural landmarks to align scan positions.

### 2.4 Optical Safety Limits and Considerations

Acquisition settings and output power for RS and OCT were recorded for MPE calculations, following ANSI Z136.1 standard formulas. Similarly, an infrared camera was used to determine if any tissue heating was induced by the OCT or RS scans and further described in the supplementary information.

## 3 RESULTS

### 3.1 Clinical Feasibility of Raman Spectroscopy in the Middle ear

Raman spectra acquired from the middle ear *in vivo* was achieved in a cohort of 10 pediatric subjects undergoing myringotomy and TT placement. The physician’s visual diagnosis was used as the gold standard for the determination of OM, with subjects identified with either recurrent AOM (N_subj_ = 6) or OME (N_subj_ = 4). Both ears were scanned as part of this study (N_ears_ = 20). Effusion was found to be present in either: one ear (N_subj_ = 4), both ears (N_subj_ = 3), or neither ear (N_subj_ = 3). Manual positioning of the probe over different TM quadrants was not seen to affect the measured spectrum. The 3.6 mm RS spot on the TM surface effectively averaged the RS signal over a larger area of the TM, which included the malleus bone in nearly all acquisitions, in addition to obscuring the imaging field of the surgical microscope. Therefore, the four measurements taken at each quadrant were averaged within a single ear to improve the signal-to-noise ratio (SNR) for each subject. Lack of axial positioning feedback within the narrow ear canal led to difficulty in maintaining a repeatable 8 mm offset from the TM. As a result, a subset of saturated measurements (N_ears_ = 5) were eliminated from further analysis.

**FIGURE 2 |:**
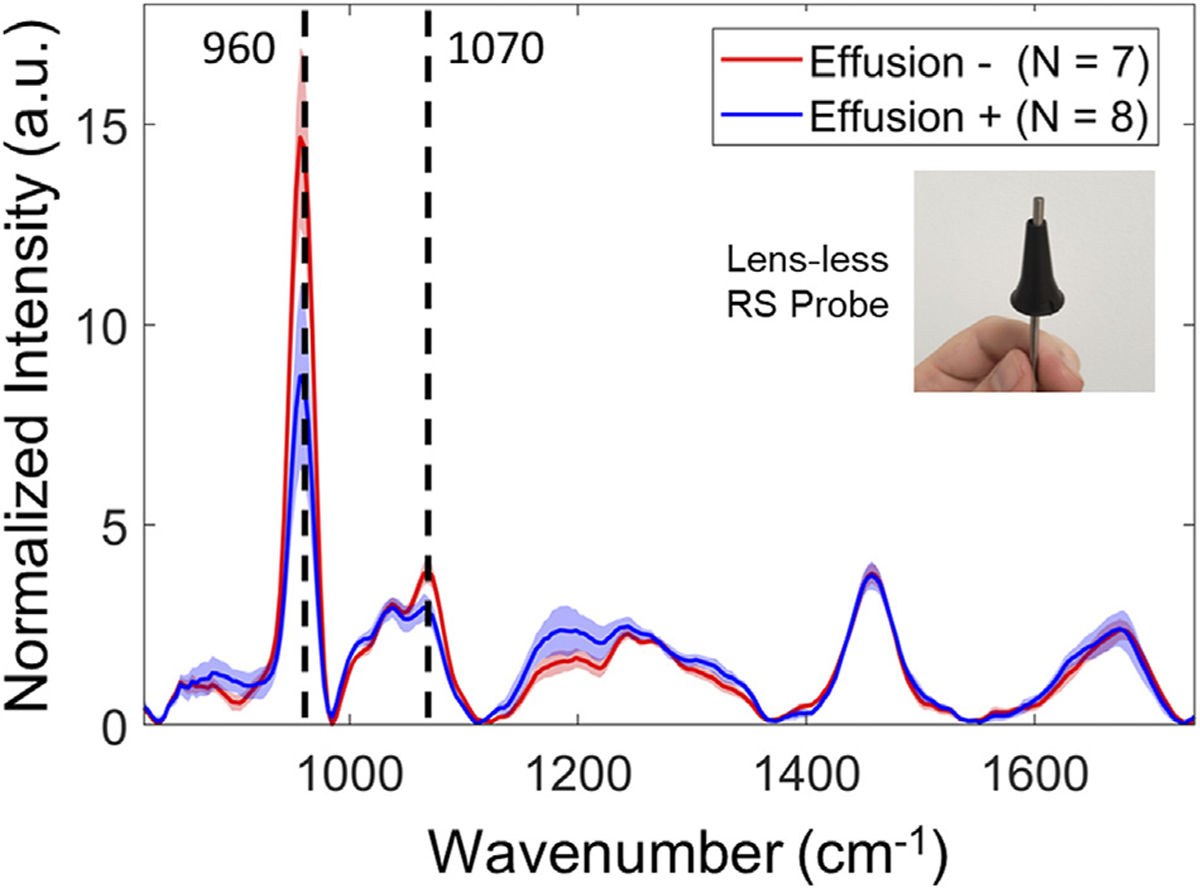
Representative clinical RS data from 10 pediatric subjects presenting with OM. Spectra were acquired with the lensless clinical probe, positioned roughly 8 mm offset from the TM. Mean normalization between 1000–1750 cm^−1^ was performed to avoid effects from the strong and highly variant 960 cm^−1^ phosphate band. Spectral plots represent the mean and 1 standard deviation, color coded for presence of effusion (Effusion +, Blue) and dry ears (Effusion −, Red). A notable decrease in bone-related Raman bands at 960 and 1070 cm^−1^, indicated by the dotted lines, was seen between ears with effusion and dry ears.

The results from clinical RS measurements (N_ears_ = 15) are shown in Figure 2. The spectra exhibit strong Raman mineral peaks, indicated by dotted lines, located at 960 cm^−1^ (v_1_-PO_4_ symmetric stretching) and at 1070 cm^−1^ (v_1_-CO_3_ symmetric stretching) that are indicative of bone (Morris and Mandair, 2011). Spectra were intensity-normalized to the mean value of the region between 1000–1750 cm^−1^ due to high variability in the 960 cm^−1^ peak. The presence of effusion is seen to influence these bone-related Raman bands, where the mean value at 960 and 1070 cm^−1^ decreased by 40 and 24%, respectively, in ears that had an effusion present (i.e., Effusion +, blue curve, N_ears_ = 8) compared to dry infected ears (i.e., Effusion −, red curve, N_ears_ = 7). In all measurements, the spectra exhibited a uniquely large proportion of non-specific background signal relative to Raman peak energy, termed signal-to-background ratio (SBR). Because detector shot noise is proportional to total signal counts (Bowie et al., 2000), this strong background signal component limits the SNR of Raman peaks. Low SBR also impacts fluorescence background fitting during RS pre-processing routines (Lieber and Mahadevan-Jansen, 2003). Bone is known to be strongly fluorescent, with the degree of fluorescence depending on the amount of mineralization and collagen within the bone matrix (Gatin et al., 2019). The bone mineral Raman bands and large fluorescent background seen in the RS signal suggests that the spectral profile measured within the middle ear is dominated by ossicles that are located directly behind the TM. These clinical results demonstrate the importance of understanding signal contributions from individual middle ear components (i.e., TM, ossicles, effusion), and their combinatory signal, to assess their impact on proving specific RS and OCT features from effusion or biofilm when measured *in vivo*.

### 3.2 Characterization of Isolated TM With Raman Spectroscopy and Optical Coherence Tomography

**FIGURE 3 |:**
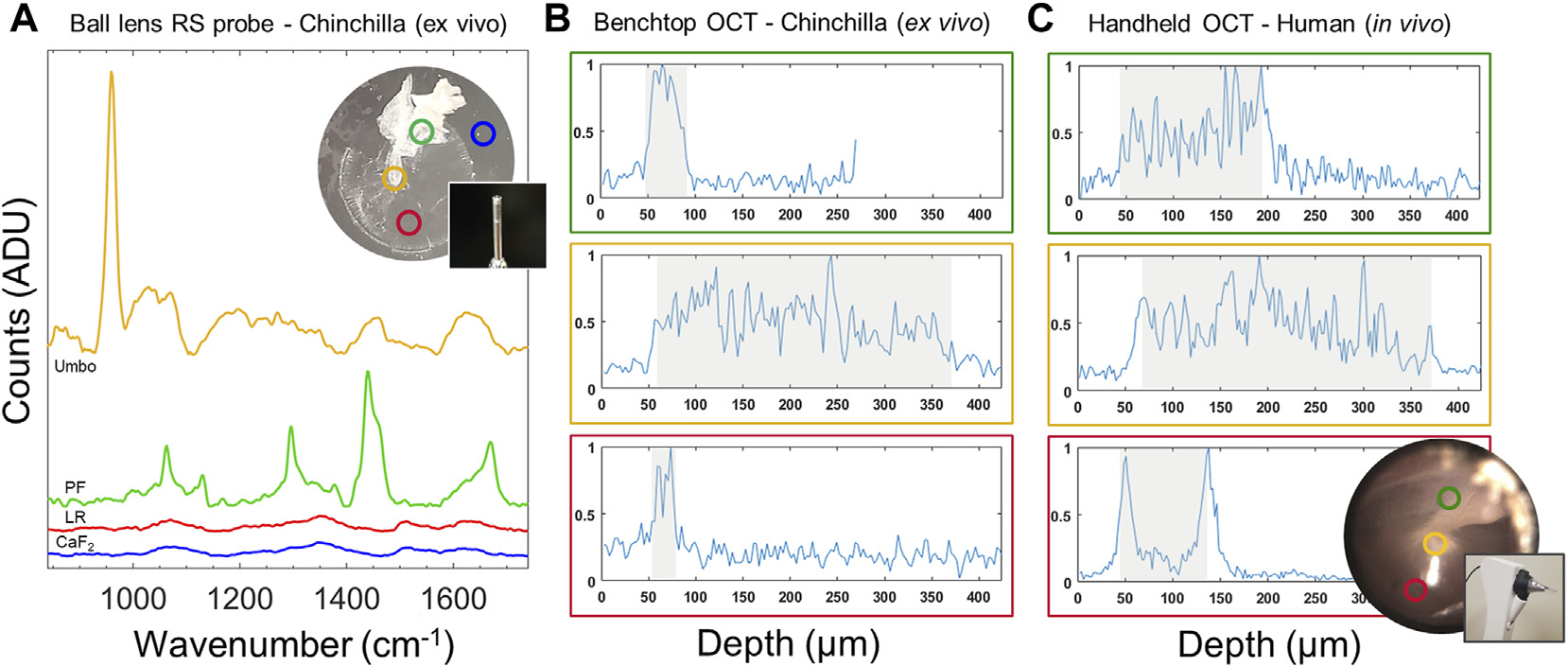
Characterization of TM for spatially dependent spectral and imaging features. **(A)** RS signal acquired using a ball-lens probe from sample substrate (Blue), the light reflex region (Red), umbo (Yellow) and pars flaccida upper region (Green) across the *ex vivo* chinchilla TM with ball lens probe; spectra offset for clarity**. (B)** Benchtop OCT depth-profiles of the chinchilla TM from the same locations as in **(A)** demonstrating structural differences. The Pars flaccida region (Green) is thicker (~ 55 μm) than the TM at the light reflex (~ 25 μm). **(C)** OCT depth-profiles from equivalent regions from a healthy adult human TM. Shaded regions in B and C denote a manually labeled thickness of relevant structures. A typical human TM at the light reflex has a baseline thickness of approximately 90 μm as shown, with thicker upper regions compared to the chinchilla TM. OCT Y-axis show arbitrary normalized intensity units. PF, Pars flaccida; LR, Light reflex; CaF2: (substrate).

To investigate the basis of TM-related spectral signatures that were observed during *in vivo* middle ear RS scans, an *ex vivo* isolated chinchilla TM was characterized and imaged with RS and OCT (Figure 3). Spatially specific Raman spectra were collected with a ball lens RS probe placed in near contact (<1 mm) with the tissue and 2-D OCT scans were acquired across the same measurement positions. The spectral plots (Figure 3A) are color-coded to show representative spectra, offset for clarity, from the CaF_2_ substrate (blue), and major regions around the TM: light reflex (red), pars flaccida (green) and umbo (yellow). Non-normalized data in analog-to-digital units (ADU) is plotted to provide a visual comparison of the relative Raman scattering intensity at each region. Compared to other TM regions, the measurement from the light reflex region has minute signal nearly equivalent to the substrate, as the RS signal intensity is proportional to tissue layer thickness. This may explain why thicker regions like the pars flaccida region has a relatively greater signal intensity (Velson et al., 2020).

Benchtop OCT A-scan data extracted from 2-D scans for each TM region (Figure 3B) are plotted to show equivalence to *in vivo* OCT scans of a healthy adult human TM (Figure 3C) taken with a handheld probe, described later in this paper. It can be observed that the extracted chinchilla TM within the benchtop model is much thinner than human TM at the light reflex region (~25 vs. ~110 μm) as highlighted in grayed regions in Figure 3B,C. Nonetheless, the structural characteristics of the TM from each species are comparable. Specifically, the unique structural characteristics of the light reflex region (Red) is identified by two strong peaks characterized by the TM, the outer (ear canal—> TM) and inner (TM—> middle ear cavity) membrane interfaces. Scanning over different regions can interrogate the umbo (Yellow)—TM and malleus bone, or the thicker connective tissue in the upper pars flaccida region (Green). Apart from structural interrogation, OCT scans provide the necessary positioning feedback in depth to ensure a consistent probe-tissue offset, as well as lateral positioning over the thin and Raman-silent light reflex region.

### 3.3 Characterization of Middle ear Component Model With Raman Spectroscopy and Optical Coherence Tomography

**FIGURE 4 |:**
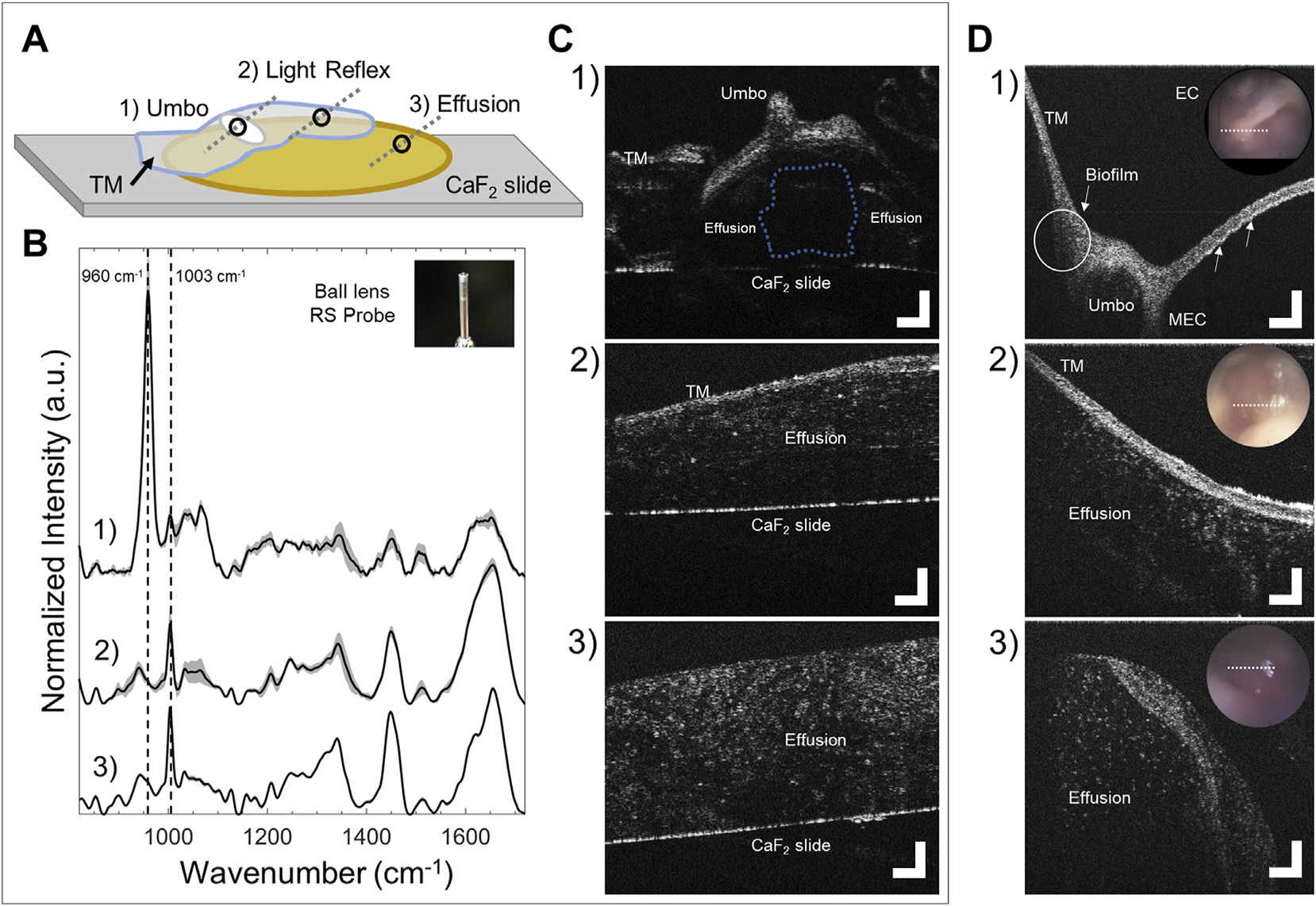
RS and OCT analysis of *in vitro* infection model, and comparison to representative pediatric human OM data. **(A)** Diagram showing the model configuration. A chinchilla TM with attached malleus bone (translucent blue and white oval) was positioned over an extracted human effusion (yellow region). Black circles and gray dotted lines denote RS and OCT scan locations, respectively. 1) Umbo over effusion, 2) TM (light reflex) over effusion, and 3) effusion alone. **(B)** Normalized RS spectra from the *in vitro* model across different sites taken with the ball lens probe (mean + one standard deviation of three measurements). Dotted lines indicate the 960 cm^−1^ (phosphate) and 1003 cm^−1^ (phenylalanine) peaks. **(C)** Benchtop OCT cross-sectional scans from the *in vitro* model taken at identical sites to RS scans. (D) Representative *in vivo* handheld OCT cross-sectional scans from pediatric OM (cited in main text) demonstrating comparable OCT image features as **(C).** D_1_: OCT image across the umbo showing biofilm adherence on the left of the bone (white circle), and an additional bright scattering layer adhered to the TM (white arrows). D_2_: Subject with AOM and purulent effusion. D_3_: Purulent effusion that erupted from the TM immediately after myringotomy. TM–tympanic membrane; EC–Ear canal; MEC–Middle ear cavity. OCT scale bars represent 250 μm.

With a better understanding of the influence from the TM on RS and OCT data, a clinically relevant model was created by combining multiple middle ear components. This *in vitro* model was composed of a thawed human effusion sample and a TM extracted from a chinchilla. Figure 4 shows a diagram of this model and representative RS and OCT data.

Benchtop cross-sectional OCT scans (Figure 4C) were taken from three locations across the model as observed previously in Figure 3: over the pars flaccida, umbo, and light reflex. Although a human’s overall size is much larger than the chinchilla, the scans are comparable in terms of morphology. *In vivo* pediatric OCT data collected from prior studies (Monroy et al., 2017; Monroy et al., 2018; Monroy et al., 2019) are shown in Figure 4D for comparison. In spot 1), the umbo, inverted in the *in vitro* model, is notably smaller in the chinchilla ear, though has similar structure to a human (Figure 4D_1_). A small air bubble is trapped in the model here in between the effusion (Figure 4C_1_, blue dotted outline). In spot 2), the light reflex region in both scans (Figure 4C_2_, 4D_2_) look visually similar, though as seen previously, the chinchilla TM is thinner than the human TM. For spot 3), The thawed effusion in Figure 4C_3_ was not impacted visually from freezing, and appears with similar texture and particulate distribution to a fresh effusion from a previous study that erupted out of the TM after myringotomy (Figure 4D_3_). Apart from these morphological assessments, OCT can provide clear guidance for RS to target the light reflex area and avoid the ossicles during signal acquisition, in addition to providing an accurate probe-tissue offset.

Each RS plot (Figure 4B) represents the mean and one standard deviation of three normalized Raman spectra acquired after removing and replacing the ball lens probe in near contact (< 1 mm) over each region scanned with OCT. Spot 1) over the umbo created a spectral profile similar to the *in vivo* case that showed low SBR due to the presence of the strong ossicle spectral bands. Spot 2) interrogates the region where the TM light reflex overlaid the human effusion. The data here was consistent with the pure effusion spectrum in Figure 3, validating that the light reflex region of the TM does not substantially contribute a Raman signal. Dotted lines in the plot for Spot 2) outline the elimination of phosphate band at 960 cm −1 and emergence of 1003 cm^−1^ peak, as the RS probe is positioned away from the umbo. Spot 3) measures a region of human effusion. The 1003 cm^−1^ peak is a characteristic spectral feature associated with effusion (Pandey et al., 2018). These assessments give biochemical relevance to the observed structures seen in OCT images from this model.

Together, RS and OCT demonstrates a clear advantage to collect high quality data. Assessment of tissue using both morphological and biochemical information, along with more precise targeting of the light reflex region and a reliable probe-tissue offset, helps avoid any interference from bone.

### 3.4 Design Considerations for a Handheld Raman Spectroscopy-Optical Coherence Tomography Probe Prototype

**FIGURE 5 |:**
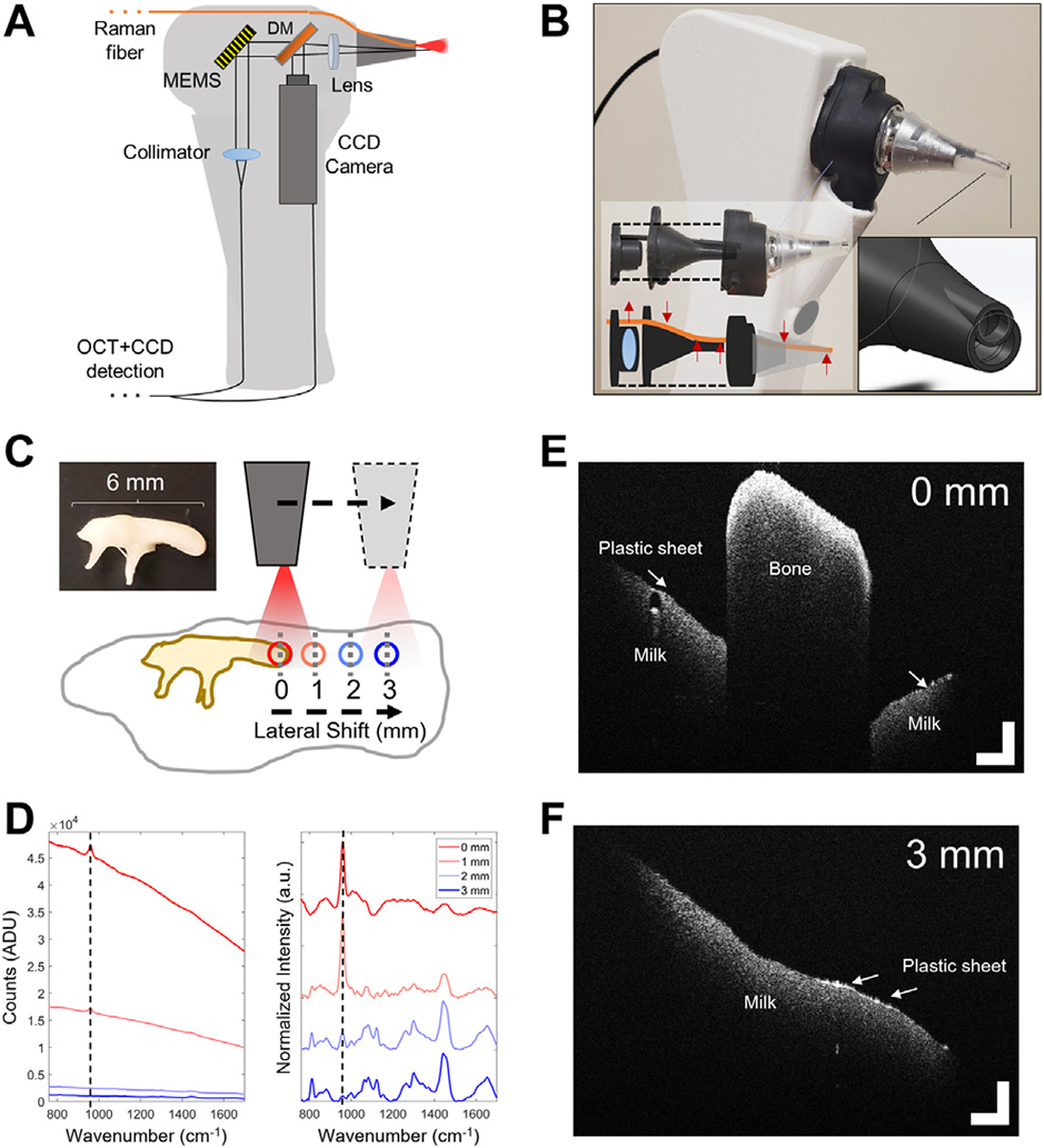
Feasibility testing of a combined RS-OCT handheld device. **(A)** Optical schematic and layout. **(B)** Modified imaging head accepts the RS1 fiber probe. Small ports were added to the design that allow the RS fiber to securely transit the otoscope head and speculum with minimal bending. **(C)** *In vitro* ear model composed of a chinchilla malleus bone suspended on top of 2% milk solution by a thin plastic wrap. RS-OCT signals acquired while laterally shifting the speculum away from the malleus by 1–3 mm. **(D)** Left: Uncorrected Raman spectra from various shifts, color coded for shift position. Right: Pre-processed normalized spectra, offset for clarity. Dotted lines denote 960 cm^−1^ phosphate band related to bone matrix. OCT scans from **(E)** 0 mm offset from malleus, and **(F)** 3 mm offset from malleus over the plastic sheet and milk solution. DM, Dichroic Mirror; CCD, Charge-coupled device color camera; MEMS, Micro-electromechanical system 2-axis scan mirror. OCT scale bars represent 250 μm.

While validation of imaging and spectral features from middle ear components was done using separate RS and OCT systems, any future clinical applications require a single user-friendly device. A multimodal handheld probe was developed to address the challenges associated with integrating both modalities in a clinically relevant design. A mockup and prototype of this handheld probe is shown in Figure 5. In this current design, the imaging head of the probe was modified to allow the lens-less RS fiber probe, previously used to acquire clinical RS data, to ultimately sit at the tip of the otoscope speculum, oriented towards the TM (Figure 5A+B). Small port openings were added in the otoscope head, denoted by red arrows in the figure, that help support the RS fiber and ensure proper alignment to the tissue target. This also prevents severe bending of the fibers to minimize optical losses. The speculum was similarly re-designed to include a structural support ring for the RS probe to ensure its alignment remained in place during acquisition, as shown in Figure 5B. The RS probe and plastic support ring in the speculum occlude 19% of the speculum opening at the top of the frame, although OCT scan performance was not impacted. Both the handheld and benchtop OCT configurations utilize equivalent components (objective lens, collimator, MEMS scanner, etc.) and thus have equivalent imaging performance, including axial and lateral resolutions of 2.3 and 35 μm, respectively. The OCT scan beam is set to collect 2-D cross sectional scans and centered in the speculum. The lateral OCT scan range on the handheld was limited by the outer diameter of the otoscope speculum to ~4 mm. In this arrangement, the probing beams are aligned such that the ~3.6 mm RS spot excites the tissue region being imaged within the OCT scan range. With this system, RS and OCT scans can be acquired sequentially. The RS probe tip was positioned approximately 8 mm away from the tissue surface, such that the RS data was collected at a similar offset to that used in the clinical scans.

To examine the performance of this integrated handheld probe design, a simple middle ear model was created by combining a chinchilla malleus bone, thin plastic wrap to act as the TM, and a 2% milk solution to represent the desired optical and RS properties of a human effusion. Although the chinchilla TM and human effusion sample was unavailable for this test, milk provides a similar biological RS spectral profile and Raman scattering cross section to that of a middle ear effusion. A comparison is shown in Supplementary Figure S1. The integrated RS-OCT device was then held in-hand over this model at a probe-tissue offset of 8 mm with identical laser power and acquisition settings used to acquire clinical RS measurements. OCT imaging was used to examine the model structurally, and to guide the position of the RS probing beam to orient it away from the malleus and maintain the desired probe-tissue offset. The malleus bone was suspended atop the milk solution by transparent plastic wrap. RS spectral measurements were acquired from over the top of the malleus, and then at 1–3 mm lateral shifts from this position (Figure 5C). As the RS-OCT device is positioned away from this bone using real-time OCT imaging, the autofluorescent background generated from the malleus is gradually reduced (Figure 5D-left), and biological Raman bands related to milk become resolvable, while the 960 cm^−1^ peak relating to bone diminishes (Figure 5D-right). This experiment demonstrates that OCT imaging can provide both microstructural information about the middle ear model and guide the positioning of the RS-OCT probe to avoid spectral interference from the ossicles (Figures 5D+F).

### 3.5 Maximum Permissible Exposure Considerations

Maximum permissible exposure (MPE) and system exposure calculations for all configurations of both RS and OCT systems used in this study are detailed in Table 1. For RS, based on the excitation wavelength and power incident on the target (785 nm/45 mW), calculations were performed for both probes (lensless and ball lens). For OCT, (λ_c_ = 860 nm, 2.35 mW at sample arm) non-scanning and scanning configurations were calculated. The ANSI equations and tables (ANSI Z136.1-2014) for skin were used as the closest analogue to the TM, as no specific tables for the ear exist. The approach taken in similar studies in literature were noted (Cain et al., 2006; Zhao et al., 2008; Oliver et al., 2013; Schulmeister, 2017; Varkentin et al., 2018). Overall, the calculated system exposure levels in this study were below MPE limits. With an effusion or other infectious components within the middle ear during OM, the fluid or biofilm should act as a thermal buffer and further reduce the risk of any thermal injury.

## 4 DISCUSSION

This paper focuses on understanding key design criteria needed for successful application of RS-OCT for OM diagnostics. Challenges in measuring probe-based RS signals within the middle ear were identified and addressed. Middle ear tissues were characterized with RS-OCT on the component-level and in a combined *in vitro* model. Ultimately, the light reflex region of TM emerged as the optimal position for interrogating imaging and spectral features of OM infection. Not only is the light reflex easily identifiable when observing the middle ear, but most importantly the degree of spectral interference from the TM or ossicles is minimal at this location, allowing for unhindered interrogation of any infectious components (e.g., effusions, biofilms). Lastly, a combined RS-OCT probe was constructed that demonstrates the benefits of a multimodal approach and clarified design constraints crucial for developing a future device.

In the clinical *in vivo* human RS study (Figure 2), the unexpected impact of the bone spectral bands and autofluorescence was discovered. An overwhelming proportion of the Raman spectral contributions came from bone mineral peaks located at 960 cm^−1^ (v_1_-PO_4_ symmetric stretching) and at 1070 cm^−1^ (v_1_-CO_3_ symmetric stretching) (Morris and Mandair, 2011). Bones create an interfering Raman signal that impedes characterizing OM infectious components, and are known to generate markedly higher autofluorescence compared to other tissues (Shah, 2020). Subsequent investigation of the malleus reveals that middle ear ossicles generate a significantly higher level of detected fluorescence compared to other bones, shown in Supplementary Figure S2. The large spot size of the diverging RS source when illuminating the TM at an 8 mm offset (e.g., ~3.6 mm diameter on surface) means that the RS probe must be carefully positioned in order to avoid the ossicles, which would otherwise overshadow the weaker Raman signal relating to tissues of interest to characterizing OM (e.g., effusions, biofilms). The associated bone Raman bands at 960 and 1070 cm^−1^ decreased in ears with effusion relative to the measurements from dry ears. This effect is likely because effusion any present will scatter a proportion of the illumination light before reaching the ossicles and causes a similar attenuation effect on the Raman photons generated by the ossicles from being detected.

The RS scans of an *ex vivo* chinchilla TM (Figure 3A) clearly demonstrate the spatial dependence of the RS signal from different regions of the TM. The light reflex region (Red circle) generates very little signal and is evident by comparison to the substrate (Blue circle). Given the translucent appearance and relatively thin profile, the scattering cross-section of the TM at the light reflex is small, which is confirmed in Figure 3B with OCT. A unique RS signature was noted from the pars flaccida (Green circle), showing distinct spectral features at 1063 and 1128 cm^−1^ (C-C stretching), 1296 cm^−1^ (CH_2_ twisting) and 1437 cm^−1^ (C-H bending) that are indicative of lipid acyl chains (Krafft et al., 2005) and phospholipids (Czamara et al., 2015). A broader Amide I band (1590–1720 cm^−1^) is also seen that relates predominantly to proteins and connective tissue like collagen. The umbo, where the malleus attaches to the center of the TM (Yellow circle), generated a spectrum with a strong bone-related peak at 960 cm^−1^ and substantial fluorescent background that was similarly noted during clinical RS scans of the middle ear. This confirms that a large proportion of the undesirable signal acquired *in vivo* arises from the ossicles, and that the light reflex is the optimal location to acquire Raman spectra of middle ear biofilms and effusions. The light reflex region is a simple visual target to aim for, as it best avoids addition of TM Raman signal present in thicker connective tissue, and is physically separated from the umbo that introduces ossicle-related spectral interference. OCT scans are similarly best taken from the light reflex to ensure consistent scans between subjects. With OCT, The light reflex region is characterized by two clearly defined peaks with a small separation dependent on the thickness of the TM. Within an integrated RS-OCT device, these OCT image features would ensure the RS probing beam is properly laterally positioned over this region during *in vivo* scans and provide axial positioning feedback on the probe-tissue offset, in addition to any positioning cues from the integrated CCD camera. Depth profiles of the *ex vivo* chinchilla TM (Figure 3B) and in vivo adult normal human ear (Figure 3C) are comparable, apart from the obvious difference in size between species.

The reported *in vitro* ear model (Figure 4) was employed to determine the Raman spectrum of pure human effusion and the effect of intermixed signals from middle ear components. The degree of spectral interference caused by various middle ear tissue is shown in Figure 4B. Spot 1) in the *in vitro* model, measuring umbo over effusion, created a spectral profile similar to the *in vivo* clinical data (comparing Figure 4B_1_ to Figure 2). Strong bone spectral bands and low SBR in this measurement indicates the presence of malleus remnants still adherent to the umbo, which creates difficulty in resolving effusion spectral features. Spot 2) in this *in vitro* model, measuring the light reflex over effusion, was consistent with measurements from spot 3) that represented pure human effusion. This confirms the light reflex as an ideal location to probe effusion biochemical composition *in vivo* with RS.

To confirm the reported effusion spectral line shape, a Raman spectrum from the same effusion sample was subsequently scanned on a Raman microscope (Renishaw, United Kingdom) system (Supplementary Figure S3). The effusion spectrum was validated and is in general agreement with previously published microspectroscopy data of human effusion (Zhao et al., 2016). OCT scans of the *in vitro* model in these three regions (Figure 4C) are comparable to previously observed *in vivo* scans of pediatric OM (Monroy et al., 2017; Monroy et al., 2018; Monroy et al., 2019), Figure 4D, particularly at spot 2) over the light reflex region. Dense mucoid fluid in panel 4C_3_/D_3_ appear as point scatterers in a turbid media. Effusions often show these characteristic patterns depending on the relative ratio of infectious components and particulates (mucous, bacteria, immune cells, etc.) as observed in many prior studies (Won et al., 2018; Monroy et al., 2019). Therefore, the selected clinical scans are representative of severe cases of OM that present with a mucoid, dense fluid and little inflammation of the TM, and demonstrate the equivalence of the *in vitro* model used here. Ultimately, these image features provide guidance for proper placement and orientation of the probe, to ensure sampling over the light reflex and away from the ossicles such that high-quality RS scans can be captured.

A prototype RS-OCT handheld device was constructed and tested on a simple model composed of malleus bone suspended over milk solution by a thin plastic sheet. OCT imaging provided both depth and lateral positioning feedback to control the probe-tissue offset and avoid the malleus, which interferes with detecting Raman features from the milk (Figure 5D). Characterization of this model with RS-OCT is straightforward due to its relatively homogenous composition (Figure 5E+F). The thin plastic sheet representing the TM is not as clearly visible in some regions due to its limited thickness and the observed high scattering content of the milk, a colloidal solution of fats and protein in water. The malleus can be easily detected by either its structural or spectral characteristics. By appropriately positioning the handheld device over the simulated light reflex, the characteristic spectra of milk was accurately collected (Silva et al., 2021) without any interference from the malleus. In summary, this simplistic model confirmed the benefits afforded by the multimodal probe that is expected for improved signal acquisition within the middle ear.

MPE limits were evaluated using tables available for skin. Total system exposure for the handheld device meets safety guidelines in this configuration, which is an important consideration for future clinical translation. Infrared thermal imaging shown in Supplementary Figure S4 corroborates these MPE calculations, demonstrating that there was minimal heating on human skin (hand/finger). MPE is related to energy per unit area per unit time as well as the local tissue properties, particularly the absorption and scattering properties of the tissue and the heat capacity. Given the transparent nature of the TM and the substantially thinner profile than bulk skin tissue, we expect less absorption and heating effects than in typical skin.

Overall, multimodal RS-OCT provides a wealth of diagnostic information that has the potential to improve the assessment of OM *in vivo*. Morphological and compositional changes in the ear caused by any infectious components (effusion, biofilm, etc.) can be fully characterized. For example, when in position over the light-reflex region, OCT can characterize the structural distribution of biofilm and effusion properties and control the probe-tissue offset. RS can then accurately probe biomolecular information from components of interest that are present behind the TM and avoid interfering signal from ossicles. Together, the strengths of each technique complement their individual weaknesses. RS-OCT captures a direct link between the observed structural image properties (texture, thickness, etc.) of tissues within the middle ear and their biochemical signatures.

### 4.1 Study Limitations

Experiments performed in this study examined important use cases and realistic middle ear models to evaluate the ability of RS-OCT to assess OM. Many crucial operational parameters were better understood, namely: lack of substantial RS signal contribution from the TM over the light reflex region, an aberrant signal from the ossicles, and verification of safe system exposure levels (MPE). However, the initial findings for *in vivo* measurements prompted an in-depth evaluation of components as in the *in vitro* model. Samples were frozen and thawed before interrogation or were from a normal healthy chinchilla TM. In future studies, fresh (i.e., not frozen) human samples or *in vivo* measurements will be used to correlate RS-OCT data to the presence of specific bacterial species as well as identify any effects of inflammation. To address some of the shortcomings of the current optical design, a future integrated design is being conceived that considers more effective RS and OCT imaging configurations, and an optimized speculum tip with an equivalent outer diameter to standard pediatric tips.

### 4.2 Design Considerations for Future System Development

Development of the RS-OCT handheld prototype fulfills many practical considerations for clinical imaging of the ear. During testing, it provided unique insight into improvements for a next-generation system. The dimensions and conformation of the ear canal impose the least flexible set of parameters in this application. For example, a wide-field imaging camera is paramount for visualization of the TM and desired by physicians for regular use in a clinical setting. However, many details can be gleaned from single OCT depth-profiles rather than cross sectional images, including positioning feedback for controlling RS probe-to-tissue distance. Moving to a non-scanning OCT configuration would reduce device complexity and simplify integration of both RS and OCT probes within the small speculum tip. Machine learning tools can be used to supplement the transition to 1-D OCT profiles (Monroy et al., 2019). Similar considerations exist for balancing RS sensitivity and MPE limits. Contact-based RS measurements on the TM risks perforation, damage to the ossicular chain, and extreme pain for the patient. Thus, any RS interrogation of the middle ear space must be conducted at an offset from the tissue surface.

In future designs, it would be advantageous to characterize the performance of an OCT source with illumination spectrum that falls outside of the spectral detection range of the RS spectrometer, which currently overlap in the presented system (e.g., 785–925 nm). Spectrally separated OCT and RS illumination and detection bands would allow for simultaneous RS-OCT acquisition. Equivalently, spatial constraints require components to comfortably fit within the pediatric ear canal. Unless anesthetized, it is challenging for a pediatric patient to sit still for more than a few seconds. Higher RS illumination power and shorter integration times that still meet MPE limits or novel signal collection methodologies may be needed to ensure acceptable signal quality and spatial specificity within awake children. Primary care is also a very cost-sensitive clinical discipline. Therefore, reduction in system cost, complexity and performance must be carefully weighed against imaging performance and sensitivity (Patil et al., 2011a; Emmanuel et al., 2021; Song et al., 2021) for effective detection of OM.

## 5 CONCLUSION

This paper investigated many of the operational parameters needed to advance RS-OCT for OM detection. First, the feasibility of collecting Raman spectra from the middle ear *in vivo* was demonstrated. Next, benchtop RS-OCT was used to characterize the TM alone and then within an *in vitro* model using a real human effusion. It was found that ossicle-related spectral signatures overwhelm the acquired Raman signal and burden the analysis of spectral bands arising from infectious components. Positioning the RS probe at the light reflex was most effective for recovering spectra from effusions, as this thin region of the TM produces a reasonably low Raman signal and is physically separated from the malleus bone. Cross-sectional OCT scans from these models demonstrate clear resemblance to *in vivo* scans of the human middle ear. Likewise, this study demonstrated the potential for OCT to be used to not only examine morphological information about middle ear components (e.g., TM, presence of effusions, biofilms, thickness, texture, etc.) but also to guide the positioning of the RS probe and avoid the impact of bone signals. Finally, an integrated RS-OCT handheld probe was developed and successfully investigated the feasibility and translational potential for these optical techniques to be integrated into a single device. An analysis of optical power considerations in regards to MPE limits showed systems used in this paper to be within safety limits and ready for future *in vivo* testing. Multimodal RS-OCT imaging for OM has the potential to provide clinically relevant information, such as identifying presence of effusion or biofilms with OCT, as well as effusion properties (e.g., purulent, serous or mucoid) (Schachern et al., 2017) and causative species during active infection (Ayala et al., 2017; Shen et al., 2022) with RS. Overall, this study demonstrates that a combined RS-OCT system is advantageous and provides a more comprehensive picture of the middle ear than either modality offers alone.

## Supplementary Material

Supplementary Material

## Figures and Tables

**TABLE 1 |:** Maximum permissible exposure and system exposure limits for this study. BL, Ball lens.

Optical system	Irradiance (mW)	Wavelength (nm)	Beam diameter (μm)	Limiting aperture (mm)	Exposure (J/m^2^)	System	
						MPE (ANSI) (J/m^2^)	Exposure (J/m^2^)

OCT–static beam	2.35	860	32	3.5	5.0	34366.49	1221.27
OCT–30 FPS scan	2.35	860	32	3.5	(150 cycles)	80429.52	854.80
RS1–8 mm offset	80.00	785	3600	3.6	2.5	20458.68	19648.78
RS2–contact (BL)	45.00	785	500	3.5	2.5	20458.68	11693.03

## Data Availability

The original contributions presented in the study are included in the article/Supplementary Materials, further inquiries can be directed to the corresponding authors.
