# Supplementary material for: Multimodal Handheld Probe for Characterizing Otitis Media — Integrating Raman Spectroscopy and Optical Coherence Tomography
Source: Front Photon. Author manuscript; Available in PMC 2023 Jun 17. (PMC9720905; doi:10.3389/fphot.2022.929574)
Supplement: Supplementary Material [file NIHMS1850965-supplement-Supplementary_Material.docx]

**(Supplementary material)
Multimodal handheld probe for characterizing otitis media – integrating Raman spectroscopy and optical coherence tomography**

**Guillermo L. Monroy^1†^**  **and Sean Fitzgerald^2,3†^, Andrea Locke^2,3^, Jungeun Won^1,4^,
Darold R. Spillman, Jr^1^, Alex Ho^1,4^, Farzana R. Zaki^1^, Honggu Choi^1^,
Eric J. Chaney^1^, Jay A. Werkhaven^5^, Kevin Mason^6^,
Anita Mahadevan-Jansen^2,3,5^, Stephen A. Boppart^1,4,7,8,9*^**

**^1^Beckman Institute for Advanced Science and Technology, University of Illinois Urbana-Champaign, Urbana, IL 61801, USA
^2^Vanderbilt Biophotonics Center, Nashville, TN 37232, USA
^3^Dept. Biomedical Engineering, Vanderbilt University, Nashville, TN 37232, USA
^4^Dept. Bioengineering, University of Illinois Urbana-Champaign, Urbana, IL 61801, USA**

**^5^Dept. Otolaryngology, Vanderbilt University Medical Center, Nashville, TN 37232, USA
^6^Center for Microbial Pathogenesis, The Abigail Wexner Research Institute Nationwide Children’s Hospital, Columbus, OH 43205, USA**

**^7^Dept. Electrical and Computer Engineering, University of Illinois Urbana-Champaign, Urbana, IL 61801, USA
^8^Carle-Illinois College of Medicine, University of Illinois Urbana-Champaign, Urbana, IL 61801, USA**

**^9^Cancer Center at Illinois, University of Illinois Urbana-Champaign, Urbana, IL 61801, USA**

**^†^ These authors have contributed equally to this work and share first authorship**

*Effusion mimic for middle ear model*

The performance of the integrated RS-OCT handheld device was tested using a simple middle ear model to demonstrate the benefits of dual-modal OCT imaging with RS. While human effusion samples were unavailable for this test, cow’s milk has a similar spectral profile and Raman scattering cross section to that of a middle ear effusion. Un-normalized example spectra from both fluids are shown in **Supp. Fig. 1**, offset for clarity, measured with the ball lens RS probe under equivalent laser power and acquisition settings (50 mW illumination power at 785 nm, 1 sec. exposure time, 3 accumulations).


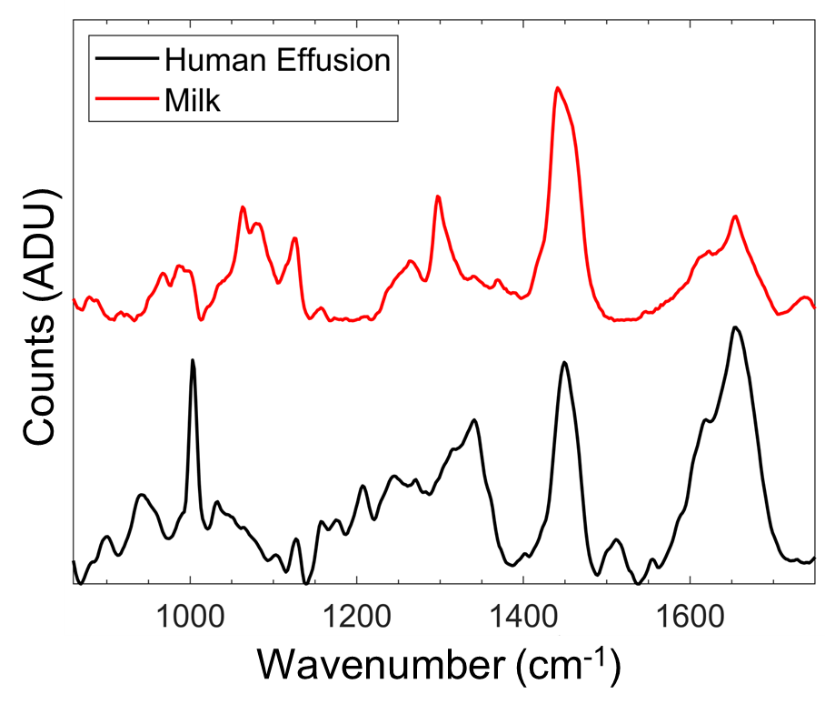


**Supp. Fig. 1**: Non-normalized Raman spectra from human effusion (black curve) and milk (red curve), offset for clarity, demonstrating the similarity in biological Raman bands between 1400-1750 cm^-1^ and general Raman scattering intensity.

*Comparison of malleus bone fluorescence*

Raman scans from the malleus bone throughout this work were noted by uniquely high background signal, which impacted spectral analysis and the SNR of Raman spectra. To investigate the relative level of background from middle ear ossicle compared to alternative bone sample, probe-based Raman scans of the malleus was compared to a tooth and tibia harvested from a rat carcass (**Supp. Fig. 2A**). Bone samples were of a similar size and thickness (i.e. 4-6 mm long and 1-2 mm thick). The clinical probe was placed in contact with both bone samples with 50 mW of power at 785 nm. Exposure time was lowered to 100 ms to avoid detector saturation. It is seen that the malleus generates ~8 times more background than the tibia and tooth samples, by a factor of 3.1 and 12.4 respectively, by comparing the integrated raw signal between 800-1800 cm^-1^. This trend was then validated by imaging these tissues with a wide-field fluorescent imaging system previously reported by our group (Nguyen et al., 2018) (**Suppl. Fig. 2B**). This system excited the imaging field with the same 785 nm diode laser used within the RS probe system and diffuse emissions were filtered with an 830 nm long pass filter (Midwest Optical Systems Inc., LP830, Illinois, USA) before being imaged by a CMOS camera (Basler AG acA1300-60gmNIR, Ahrensburg, DE); simulating the excitation and detection spectral windows used when detecting Raman spectra.

The fluorescent image confirms that the malleus does generate notably high background signal compared to tibia or tooth by a factor of 4.3 and 11.3 respectively, as measured by comparing the average image value within a region of interest manually drawn around each bone. Image analysis was performed within Fiji software (Schindelin et al., 2012). While the relative background enhancement between the samples compared in this image was close to what was seen in the Raman spectrum, this 2-D system acquires the signal with a different collection geometry than the RS fiber probe. Regardless, both trends demonstrate the unique high degree of background signal generated by malleus bone.


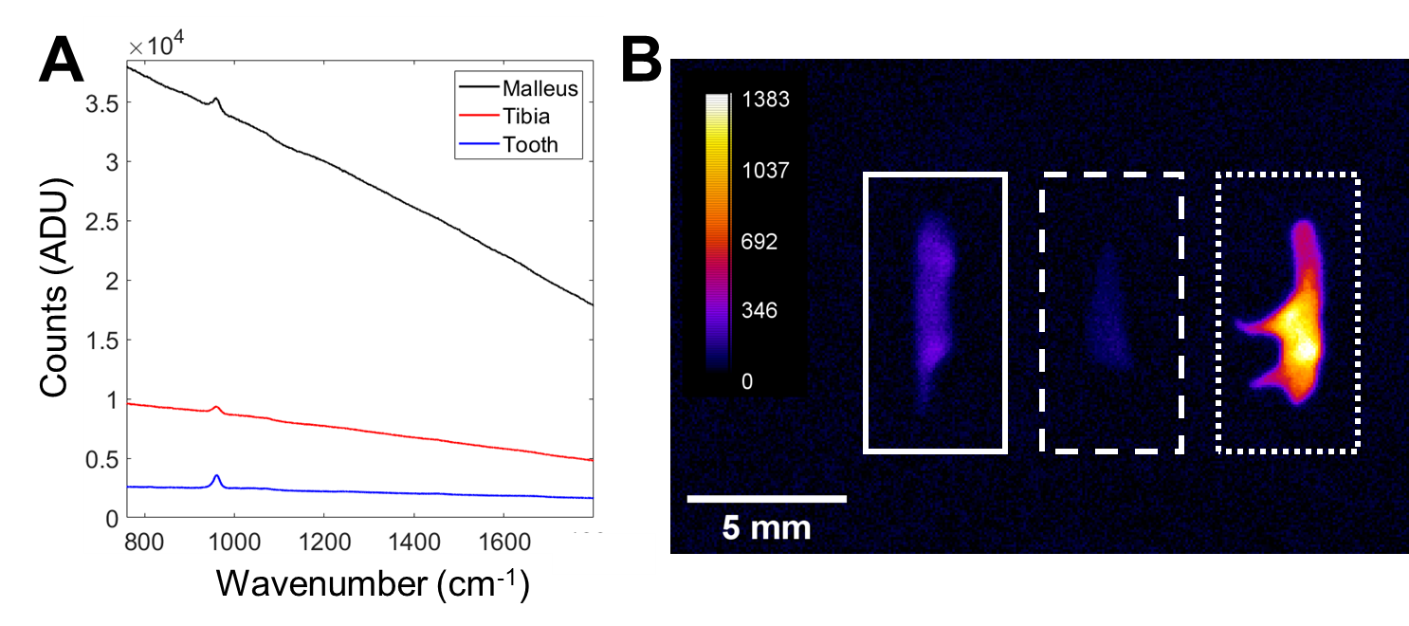


**Supp. Fig. 2**: Comparison of fluorescence generated by three rat bone samples. **A)** Raw Raman spectra from each bone, showing an increased background intensity from malleus (black curve) compared to tibia (red curve) or tooth (blue curve). **B)** Fluorescent image of the same bone samples with similar excitation and detection spectral bandwidths to that used for Raman scans. The unique increase in fluorescent counts from the malleus (right, dotted box) relative to the tibia (left, solid box) and tooth (center, dashed box) in this image validates the trend seen in the raw spectra.

*Validation of human effusion RS spectra with microspectroscopy*

The probe-based Raman spectral profiles observed for the human effusion sample were validated by measuring the same sample used within the *in vitro* middle ear model on a commercial benchtop inVia^TM^ confocal Raman microscope (Renishaw, United Kingdom). An aliquot of this effusion sample was placed in a stainless steel well to minimize substrate contributions to the acquired signal. Raman spectra were collected under a 50x objective
(NA 0.75) with 50 mW power at 785 nm and integration time of 10 sec. The spectrum agrees with data from this paper and previously published reports of RS data from human effusion (Pandey et al., 2018). However, the probe-based signal slightly differed by increased peak energies at 1340 and 1515 cm^-1^, likely due to RS excitation of adjacent components to pure effusion within this model and background contributions from the ball lens probe components. These spectra are shown in **Supp. Fig 3**.


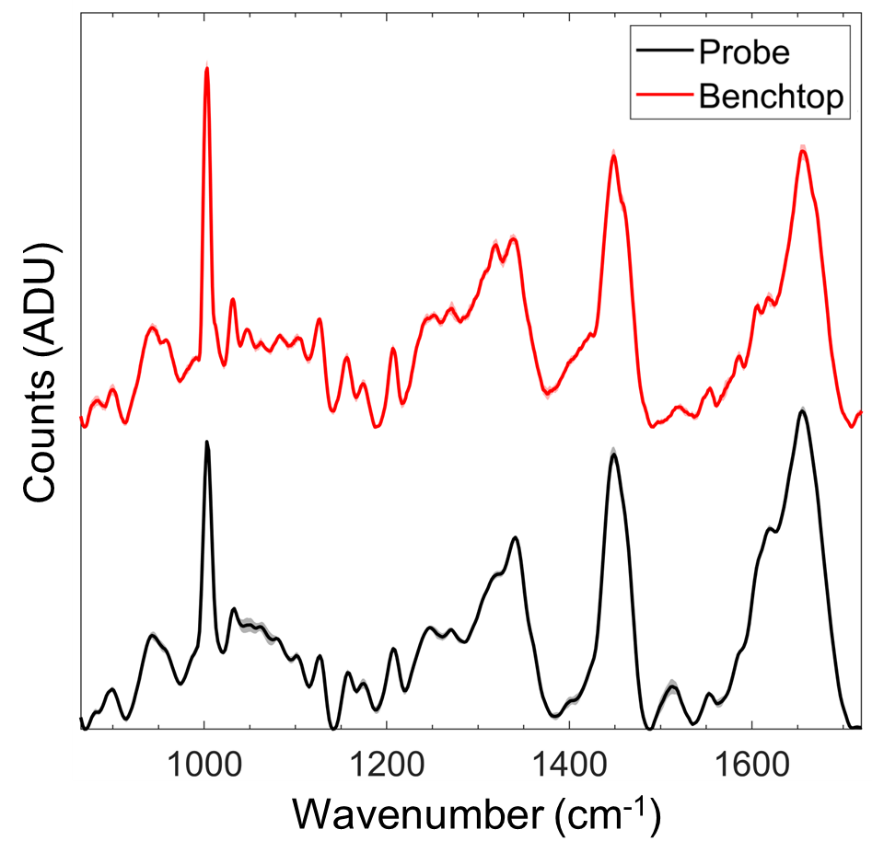


**Supp. Fig. 3**: Validation of the spectral line shape from human effusion sample measured by probe-based RS and benchtop microspectroscopy. An aliquot from the same effusion sample used in the *in vitro* model was scanned within the Raman microscope on a CaF_2_ slide. Five spectra were acquired from adjacent measurement spots on the effusion sample in both cases. Plots represent the mean and one standard deviation of these measurement sets, offset for clarity. The spectrum measured by benchtop Raman microspectroscopy (red curve) was in agreement with the probe-based spectrum (black curve) and previous reports of human effusion RS data.

*Observing potential tissue heating from RS-OCT imaging*

To experimentally observe any tissue heating effects and further validate MPE calculations, a thermal infrared camera (FLIR-T62101, Teledyne FLIR, OR) with 0.04 °C sensitivity was used to observe various targets when exposed to the RS illumination laser and OCT imaging beam under conditions used in this study. A range of scenarios was considered with results displayed in **Suppl. Fig. 4**. The *ex vivo* chinchilla tympanic membrane (**Supp. Fig. 4A**) and *in vivo* human skin (finger, **Suppl. Fig. 4B+C**) were imaged after irradiation with the light dosage parameters used to acquire RS spectra and OCT scans. As expected, there were minimal changes in temperature before and after imaging. Temperature fluctuations averaged around Δ 0.3°F, with the *ex vivo* sample in **A** dissipating heat during imaging due to the metal base and glass slide it was placed on, at ambient room temperature. The minimal heating effects observed in these tests are consistent with MPE calculations.


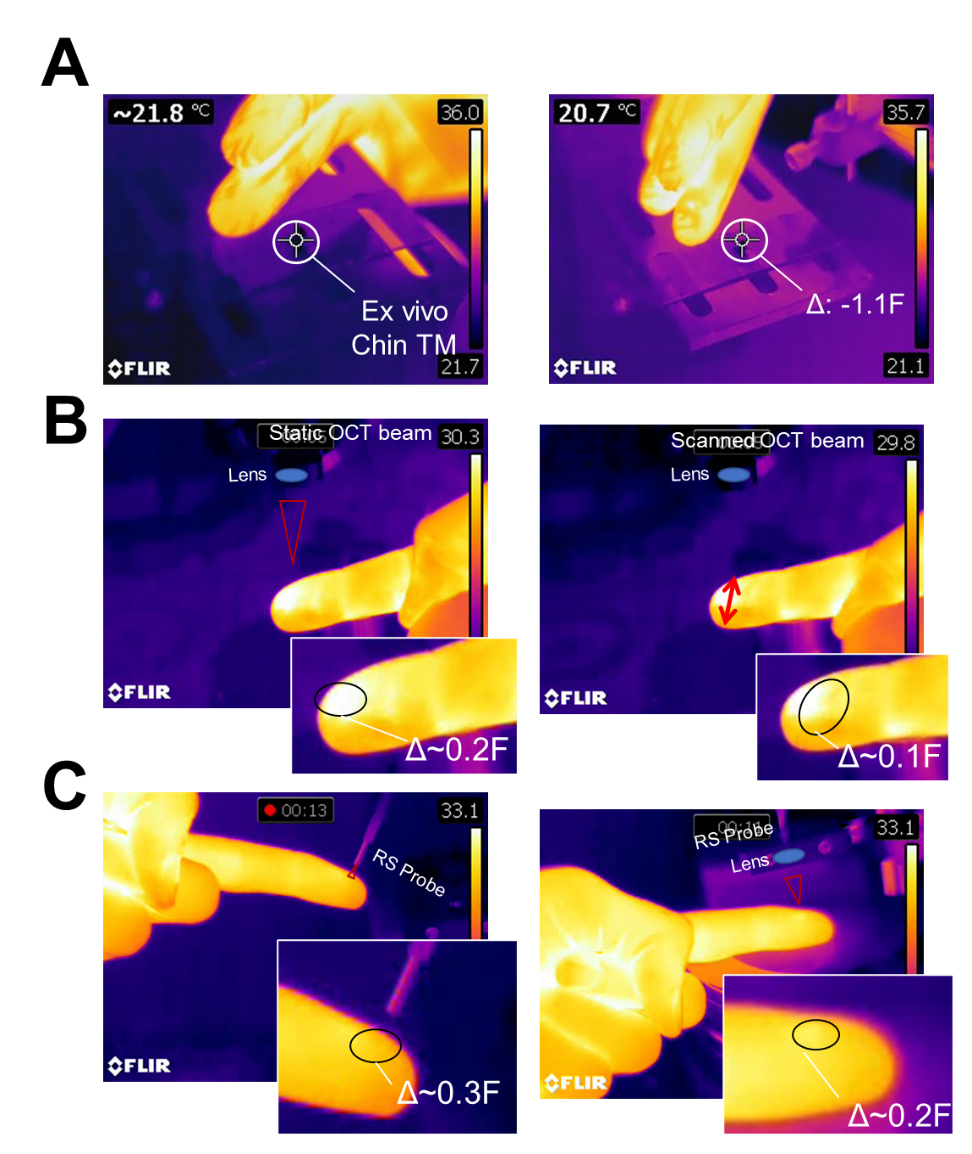


**Supp. Fig. 4**: Infrared camera images to observe for tissue heating effects during experimentation. Samples in this study were observed with a thermal camera (FLIR-T62101, Teledyne FLIR, OR) capable of measuring any temperature transients caused by RS or OCT imaging. **A)** *Ex vivo* chinchilla TM before and after RS imaging. Cooling effects from the saline and benchtop setup were greater than heating effects imparted by RS imaging. *In vivo* testing of **B)** OCT (Left: static and Right:scanned), and **C)** RS on human skin (Left: lens-less,
Right: 6 mm focal length lens). The OCT and RS probes were separated to observe heating effects. Neither technique at specified settings showed any relevant heating effects during the scans, generally confirming the safety of these techniques in bulk skin at the specified parameters as shown in Table 1 in the main text.

**References**

Nguyen, J.Q.M., McWade, M., Thomas, G., Beddard, B.T., Herington, J.L., Paria, B.C., et al. (2018). Development of a modular fluorescence overlay tissue imaging system for wide-field intraoperative surgical guidance. *J Med Imaging (Bellingham)* 5(2)**,** 021220. doi: 10.1117/1.JMI.5.2.021220.

Pandey, R., Zhang, C., Kang, J.W., Desai, P.M., Dasari, R.R., Barman, I., et al. (2018). Differential diagnosis of otitis media with effusion using label-free Raman spectroscopy: A pilot study. *J Biophotonics* 11(6)**,** e201700259. doi: 10.1002/jbio.201700259.

Schindelin, J., Arganda-Carreras, I., Frise, E., Kaynig, V., Longair, M., Pietzsch, T., et al. (2012). Fiji: an open-source platform for biological-image analysis. *Nat Methods* 9(7)**,** 676-682. doi: 10.1038/nmeth.2019.
